# Functional Connectivity in the First Year of Life in Infants at Risk for Autism Spectrum Disorder: An EEG Study

**DOI:** 10.1371/journal.pone.0105176

**Published:** 2014-08-20

**Authors:** Giulia Righi, Adrienne L. Tierney, Helen Tager-Flusberg, Charles A. Nelson

**Affiliations:** 1 Department of Psychology, University of Massachusetts at Amherst, Amherst, Massachusetts, United States of America; 2 Harvard College Writing Program, Harvard University, Cambridge, Massachusetts, United States of America; 3 Department of Psychological and Brain Sciences, Boston University, Boston, Massachusetts, United States of America; 4 Division of Developmental Medicine, Boston Children’s Hospital, Boston, Massachusetts, United States of America; 5 Department of Pediatrics, Harvard Medical School, Boston, Massachusetts, United States of America; 6 Harvard Graduate School of Education, Cambridge, Massachusetts, United States of America; Lancaster University, United Kingdom

## Abstract

In the field of autism research, recent work has been devoted to studying both behavioral and neural markers that may aide in early identification of autism spectrum disorder (ASD). These studies have often tested infants who have a significant family history of autism spectrum disorder, given the increased prevalence observed among such infants. In the present study we tested infants at high- and low-risk for ASD (based on having an older sibling diagnosed with the disorder or not) at 6- and 12-months-of-age. We computed intrahemispheric linear coherence between anterior and posterior sites as a measure of neural functional connectivity derived from electroencephalography while the infants were listening to speech sounds. We found that by 12-months-of-age infants at risk for ASD showed reduced functional connectivity compared to low risk infants. Moreover, by 12-months-of-age infants later diagnosed with ASD showed reduced functional connectivity, compared to both infants at low risk for the disorder and infants at high risk who were not later diagnosed with ASD. Significant differences in functional connectivity were also found between low-risk infants and high-risk infants who did not go onto develop ASD. These results demonstrate that reduced functional connectivity appears to be related to genetic vulnerability for ASD. Moreover, they provide further evidence that ASD is broadly characterized by differences in neural integration that emerge during the first year of life.

## Introduction

Autism spectrum disorder (ASD) is a developmental syndrome primarily characterized by deficits in social communication and interactions, and repetitive/restricted patterns of behaviors, interests, and/or activities, which are present, at least in part, from early in development [1]. The presentation of ASD is very heterogeneous, and changes depending on a child’s intellectual abilities, language proficiency, and age [2]. The phenotypic complexity of ASD has been associated with a variety of differences in both functional and anatomical neural substrates [3]. The multitude of neural atypicalities identified in individuals with ASD coupled with recent findings showing significant generic heterogeneity [4] have contributed to conceptualizing ASD as a syndrome characterized by differences in brain-wide neural circuitry that emerge across development. On the basis of this evidence, ASD is hypothesized to be a “disconnection syndrome”, one in which the anatomical and functional integration of neural circuits is disrupted [5]. Neural integration processes are reflected in various frequency domains of an individual’s EEG. High frequency activity in the gamma range, for example, is thought to bind neural information from different networks, a process that is required for a number of perceptual and cognitive tasks and that is disrupted in several neurocognitive disorders including ASD [6]. Disruptions in the binding function of gamma activity may explain a wide range of language and social communication deficits that characterize ASD [7]. More specifically, behaviors that require the coordinated function of several brain regions may not be sufficiently integrated without the appropriate amount of gamma frequency activity.

One question that has motivated recent research in this area is how early in development differences in gamma frequency metrics of neural integration arise. To investigate issues related to very early development, studies rely on infants with an older sibling with ASD [8], [9]. These infants are termed “high-risk” for ASD because they have an increased predisposition to develop ASD, estimated to be around fifteen to twenty times higher than infants with no family history of ASD [10], [11]. Studies of this population present several advantages to understanding ASD generally and to neural integration specifically [12]. First, we can ask questions about the developmental trajectories in biological and cognitive factors as they relate to the emergence of typical or atypical outcomes. Second, we can ask questions about which factors are specific to individuals who go on to develop ASD and which factors are more generally observed family members of those who have ASD. These latter factors are generally observed with greater frequency in family members of affected individuals are commonly referred to as endophenotypes or intermediate phenotypes. They form a bridge between the two ends of the causal sequence – genes and behavior [13]. Endophenotypes are particularly important to understanding ASD in that they will likely help sort through the heterogeneity that exists at each level of functionality–genetic, neural, cognitive, and behavioral [14].

With respect to neural integration as an early endophenotype, differences in gamma frequency activity have been identified in several studies of infants at high risk. For example, Elsabbagh et al. [15] found higher baseline but lower induced gamma power in response to an eye gaze paradigm in 10-month-old infants. Tierney et al. [16] found lower gamma power at 6 months and flattened developmental trajectories in high-risk infants between 6 and 24 months. Both findings are consistent with a disruption in the integration of neural networks, although the exact nature of the timing and direction of differences in baseline power still needs to be resolved. Evidence of differences in gamma power in infants at high-risk for ASD would not only provide support for the idea that ASD is a disorder of neural integration but would also provide evidence that differences of gamma activity are candidate endophenotypes.

Spectral power, however, is a limited measure of neural integration because it primarily reflects synchronized activity within the specific region in which it is measured. Other transformations of neurophysiological signals provide a better assessment of neural integration. For example, linear coherence is an index of synchronization across regions rendering it a better-suited measure of neural integration–or connectivity as it is referred to in this literature. Linear coherence assesses the correlation between the phase and power information of two EEG signals and can be applied to any frequency range. The higher the correlation, the more synchronized, and therefore integrated, the signals are interpreted to be. Indeed, studies of EEG coherence as a measure of neural connectivity have found that children and adults with ASD showed lower coherence compared to age- and IQ-matched typically developing controls [17], [18], [19]. These findings of lower coherence have contributed to the proposal that one characteristic of ASD is that there is underconnectivity between distant regions of the brain. More recent coherence studies indicate a more complex pattern of connectivity in children with ASD, finding perturbations in the proportion of long- and short-range connectivity in the theta and alpha frequency ranges [20].

Systematic studies of coherence in gamma activity in high-risk infants have not yet been conducted. If atypical patterns of neural connectivity are responsible for the symptomatology of ASD in older individuals, they are likely to emerge very early in development either before behavioral and cognitive differences emerge or concurrently with the divergence in behavior and cognitive development. If these biological indices are present early in development, they could either be biomarkers of the disorder, or like many of the other measure of neural integration, endophenotypes that are found among individuals with a high genetic load for ASD. Thus, the goal of the present study was to investigate functional connectivity in infants high-risk for ASD in order to evaluate it as a potential endophenotype or biomarker of ASD. Using coherence of gamma frequency activity as a metric of connectivity, we examined whether differences emerge during the first year of life, a period of development that precedes the onset of ASD symptoms, in infants at high or low risk for ASD as well as in the subset of those who go on to develop the disorder.

We assessed functional connectivity in the EEG signal acquired as infants were presented with speech sounds and evaluated differences in coherence in response to hearing these sounds. We employed a task that involved language relevant sounds, given that: (1) language impairments are a common feature of ASD and of the broader phenotype [21], [22] and (2) previous studies of toddlers and older children with ASD identified differences in measures of connectivity, specifically as they relate to language-based tasks [23]–[26]. This paradigm has been used to evaluate speech perception in infants [27] and previous studies of infant siblings using this task have found that there are ASD risk-related differences in the ERPs [28]. Overall we hypothesized that risk for ASD will be associated with reduced functional connectivity in response to speech sounds, which might be a manifestation of disrupted neural integration processes.

## Materials and Methods

The study reported here is part of a comprehensive and ongoing longitudinal project on the neurocognitive development of infants at risk for ASD conducted at Boston Children’s Hospital/Harvard Medical School and Boston University. All components of the study were approved by the IRB review boards at both institutions and are covered under IRB guidelines approved by both institutions. Written, informed consent was provided by the parents or guardians prior to their child’s the participation in the study.

### Participants

Participants were assigned to one of two groups in this study. If they had an older sibling with an ASD diagnosis (not due to a known genetic disorder; e.g. Fragile X syndrome), they were categorized as high-risk for ASD (HRA). The older siblings all had expert clinical community diagnoses, which were confirmed by a member of the study staff using the Social Communication Questionnaire (SCQ) [29] or the Autism Diagnostic Observation Schedule Generic (ADOS-G) [30]. Infants in the low-risk group (LRC) had at least one typically developing older sibling and no first-degree relatives with a known developmental disorder, based on a screening questionnaire. All infants had a gestational age of 36 weeks or greater, no history of prenatal or postnatal medical or neurological problems and no known genetic disorder. Furthermore, all infants were from monolingual English-speaking households (English spoken more than 80% of the time) and had no prior exposure to Bengali or Hindi.

In the present paper we report on data from 28 HRA infants and 26 LRC infants. Of the 28 HRA infants, 19 provided usable data at both the 6- and 12-month visits, 3 provided data only at the 6-month visit, and 6 only at the 12-month visit. Of the 26 LRC infants, 17 provided usable data at both the 6- and 12-month visits, 7 provided data only at the 6-month visit, and 2 only at the 12-month visit. Of the 44 infants who contributed data at 12 months of age, 38 of them were assessed using the ADOS at 36 months of age (16 LRC and 22 HRA). None of the 16 LRC infants met criteria for ASD on the ADOS (negative outcomes) whereas, 5 out of the 22 HRA infants met ASD criteria on the ADOS (positive outcomes). Expert clinical impression confirmed a diagnosis of ASD for the 5 infants with positive ADOS outcomes.

Sample demographics are presented in Table 1, displaying means for each group on characteristics of the infants and their families. There were no group differences on any demographic factors. Infants’ cognitive abilities were assessed using the Mullen Scales of Early Learning [31] at 6 and 12 months. No significant group differences were detected at 6 months of age. At 12 months of age the LRC group obtained significantly higher scores than the HRA on the Expressive Language (*t*(40) = 2.39, *p*<0.03), and Gross Motor (*t*(40) = 2.57, *p*<0.02) subscales, however the scores on these subscales for both groups were within the normal range. See Table 2 for a complete summary of Mullen scores.

**Table 1:** Mean demographic characteristic of the LRC and HRA infants (SD).

	*n*	LRC	*n*	HRA	*t*(df)	*p*
Infant’s birth weight	26	7.9(1.2)	28	7.9(0.9)	0.10(52)	0.92
Mother’s age at infant’s birth	26	33.6(4.4)	28	34.9(4.8)	−1.05(52)	0.3
Father’s age at infant’s birth	26	36.3(4.5)	28	38.2(5.9)	−1.34(52)	0.19
Mother’s education level	19	6.5(1.43)	24	5.7(1.6)	1.63(41)	0.11
Father’s education level	19	5.9(1.51)	22	5.1(2.04)	1.50(39)	0.14
Family income	19	7.4(1.61)	24	7.4(1.25)	−0.11(41)	0.91

Note that not all families provided all demographic data.

**Table 2:** Cognitive characteristics of the LRC and HRA infants at 6 and 12 months.

	LRC	HRA	Contrast
	Mean(sem)	Mean(sem)	*p-value*(df)
6 month-olds			
Visual Reception	48.6(1.8)	48(1.8)	0.816(43)
Receptive Language	49(1.3)	49.9(1.3)	0.637(43)
Expressive Language	47.1(1.1)	46.8(1.1)	0.867(43)
Gross Motor	49.1(1.7)	47.2(1.7)	0.426(43)
Fine Motor	48.2(1.6)	52.2(1.6)	0.081(43)
12 month-olds			
Visual Reception	58.8(1.9)	55.6(1.8)	0.232(40)
Receptive Language	49.6(1.8)	47.7(1.6)	0.419(40)
Expressive Language	52.3(2.1)	45.6(1.9)	**0.022**(40)
Gross Motor	48.4(2.6)	39.3(2.4)	**0.014**(40)
Fine Motor	64.5(2.1)	62.8(1.9)	0.557(40)

The scores provided are t-scores from the Mullen Scales of Early Learning. Note that a total of 4 infants did not provide data.

### Stimuli

The experimental stimuli consisted of three consonant-vowel pairs: a voiced, unaspirated, retroflex stop (/da/), native to English that represented the *standard* condition; a voiceless, aspirated retroflex palatal stop (/ta/), native to English that represented the *deviant native* condition; and a voiced, unaspirated dental stop (/dha/) not found in the English language that represented the *deviant non-native* condition. In order to allow for the matching of low level acoustic characteristics, these syllables were synthesized using STRAIGHT [32], such that all stimuli were matched on total duration (300 ms), and the two voiced, unaspirated syllables were also matched on energy, spectral components, and fundamental frequency of the vowel segment. See Seery et al. [28] for a more detailed description of the experimental stimuli.

These language-relevant stimuli have been used in previous studies examining speech perception in the first year of life. For example, it has been demonstrated that that 6 to 12 months of age is an important period in this process wherein infants become particularly skilled at recognizing speech sounds that are represented in their native language (and are thus more familiar with) while also becoming less able to recognizing speech sounds that are not represented in their native language [33], [34]. This phenomenon has been studied using ERP measures, but because it may require the coordination of auditory and higher level language areas, we investigated whether gamma coherence was sensitive to the developmental trajectories in selectively responding to native and non-native phonemes.

### Procedure

The testing session took place in a sound attenuated room. Auditory stimuli were presented using E Prime (Psychological Software Tools, PA) over two bilateral speakers while the infant sat on a parent’s lap. Each stimulus was presented for 300 ms, followed by a variable inter-stimulus interval (700–1000 ms). The experimental paradigm consisted of a double-oddball design, modeled after Rivera-Gaxiola and colleagues’ [27]. The standard stimulus was presented 80% of the time, while the two deviants were each presented 10% of the time. The experiment consisted of a maximum of 600 trials. In order to facilitate the infants’ cooperation during testing an experimenter was present in the testing room during each session. The role of the experimenter was to blow bubbles throughout the procedure, which is standard practice used to maintain the infants’ interest and increase their tolerance for the electrodes [35]. No other visual stimuli were present.

### Recording and processing of electrophysiological data

Continuous EEG was recorded using 64- and 128-channel Geodesic Sensor Nets connected to a DC-coupled amplifier (Net Amp 200, Electrical Geodesic Inc.). Data were collected from 62 of 64 and 124 of 128 possible channel locations because EOG electrodes (64-channel: 63, 64; 128-channel: 125, 126, 127, 128) that are placed on the infants face were removed from the nets to decrease fussiness. The signal was amplified with a 0.1–100 Hz bandpass filter, digitized at 250 Hz, and referenced online to a single vertex electrode (Cz). The use of two different net sizes was due an equipment upgrade that took place about two years into the longitudinal study.

Data was sampled at 250 Hz and referenced to the vertex electrode (Cz). Preprocessing of the data was performed using NetStation 4.4.2 (Electrical Geodesic Inc.). In order to prepare the data for a time-frequency analysis, the continuous EEG was segmented to 800 ms, with a baseline period beginning 100 ms before stimulus onset, and 700 ms of post stimulus onset. Automated artifact detection tools were applied to all data segments, in order to identity segments and specific channels that contained movement artifacts, eye movements, eye blinks, and off-scale activity that exceeded ±200 µV. Epochs were rejected if they contained (1) eye blinks, (2) eye movements, and (3) if more than 10% of channels in a segment were marked bad. The results of the automated artifact detection were visually inspected by a research assistant trained in the analysis of infant EEG data to confirm the presence of artifacts and ensure that all rejection criteria were applied properly. Subsequently, bad channels in all accepted segments were replaced by an automated algorithm that uses spherical spline interpolation. Finally, the data were re-referenced to the average reference.

The data were exported from NetStation into EEGLAB [36] for further processing and analysis. A 58–62 Hz notch filter was applied to remove noise created by electronic equipment present in the testing room. Subsequently, an average reference was applied to all data segments. Functional connectivity in the gamma band (30–50 Hz) was quantified as linear coherence computed using the *newcrossf* function in EEGLAB. Signal decomposition was achieved with a Morlet wavelet transform that applied 3 cycles per frequency across the frequency spectrum available in the signal.

**Figure 1:**
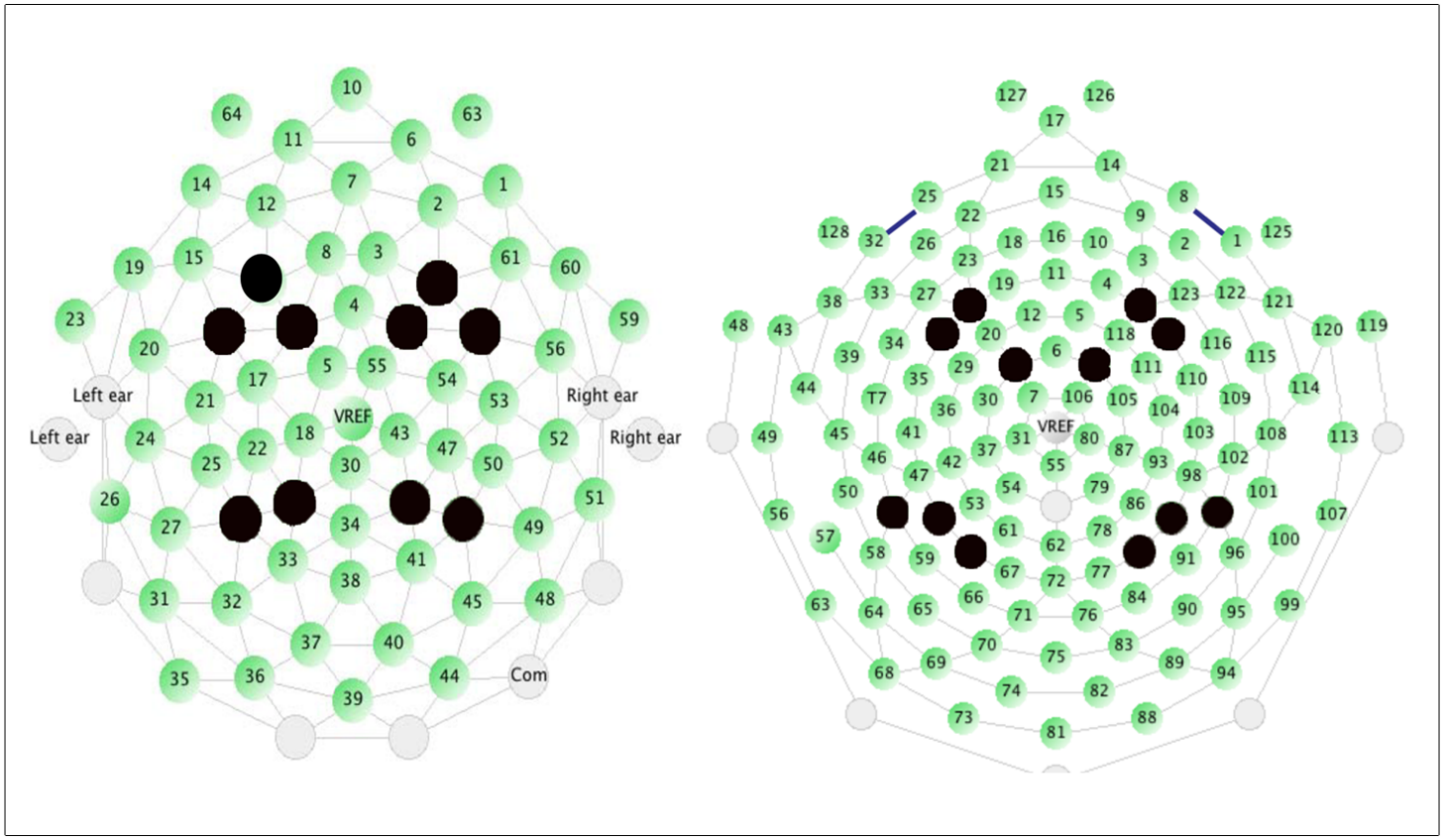
Locations of electrodes used for analysis in the 64-channel and 128-channel nets (electrodes chosen are colored in blue). Linear coherence was calculated between anterior and posterior electrode groupings within each hemisphere.

Linear coherence was calculated between electrode sites covering the frontal and temporo-parietal regions in the left and right hemispheres, and averaged across the 150 ms to 300 ms post-stimulus onset time window. This time window was chosen based on previous ERP studies that have used similar paradigms and identified components relevant to the processing of speech sounds (P150) [28]. Electrodes of interest were chosen a priori to encompass some of the anterior and posterior sites used in prior studies that have used similar paradigms [27], [28] and ensure (1) location correspondence between net types, (2) comparable skull area coverage between net types, (3) and comparable number of electrode pairs to compute average coherence between net types. The electrodes we used for each net are as follows (with the corresponding 10–10 system sites noted in parentheses). In the 64-channel net, the left frontal region contained electrodes 13 (F3), 9 (FC1), 16 (FC5); the right frontal region contained electrodes 62 (F4), 58 (FC2), 57 (FC6); the left temporo/parietal region included electrodes 29 (P1–P3), 28 (P5); the right temporo/parietal region included electrodes 42 (P2), 46 (P4–P6). In the 128-channel net the left frontal region included electrodes 24 (F3), 13, (FC1), 28 (FC5); the right frontal region includes electrodes 124 (F4), 112 (FC2), 117 (FC6); the left temporo/parietal region included electrodes 60 (P1), 52 (P3), 51 (P5); the right temporo/parietal region included electrodes 85 (P2), 92 (P4), 97 (P6). These were roughly equidistant across net types, as measured on a mannequin’s head (see figure 1).

## Results

Analyses were conducted cross-sectionally by age (6 months and 12 months) because not all infants contributed data at both time points. Repeated measures mixed-model factorial ANOVAs were used to compare group differences in linear coherence. The models included group (LRC and HRA) as the between-subject factor; hemisphere (right and left), and condition (standard, native deviant, non-native deviant) as within-subject factors; subject was treated as a random effect. Given that the number of subjects was not equivalent across the two groups, the restricted maximum likelihood (REML) method was used. The dependent measure was linear coherence. Because of the change in equipment, we also evaluated the influence of net type (64-channel, 128-channel) but found no effects; therefore this factor was excluded from the analyses reported below. Additionally, we conducted a preliminary analysis in order to compare the differences in the 12-month data between the subset of infants who at 36 months met criteria for ASD and those who did not. See tables 3 and 4 for a complete summary of the statistical analyses.

**Table 3:** Full results of repeated measures ANOVA for 6-month-old infants and 12-month-old infants.

6 months	F(df)	*p*
Group	0.07(1)	0.79
Condition	47.46(2)	**<0.0001**
Hemisphere	5.42(1)	**0.02**
Group×Condition	2.71(1)	0.07
Group×Hemisphere	0.52(1)	0.47
Condition×Hemisphere	0.96(2)	0.39
Group×Condition×Hemisphere	0.74(2)	0.48
**12 months**		
Group	7.77(1)	**0.008**
Condition	77.33	**<0.0001**
Hemisphere	1.92(1)	0.17
Group×Condition	2.01(2)	0.14
Group×Hemisphere	0.01(1)	0.91
Condition×Hemisphere	2.07(2)	0.13
Group×Condition×Hemisphere	0.02(2)	0.98

**Table 4:** Full results of repeated measures ANOVA on the group of infants who have been assessed using the Autism Diagnostic Observation Schedule (ADOS) at 36 months of age.

	F(df)	*p*
	5.8(2)	0.007
Condition	52.17(2)	**<0.001**
Hemisphere	1.08(1)	0.3
Group×Condition	1.4(2)	0.24
Group×Hemisphere	0.05(1)	0.95
Condition×Hemisphere	1.51(2)	0.22
Group×Condition×Hemisphere	0.64(2)	0.63

‘Group’ is a three-way between-subject variable: LRC infants with negative ADOS outcomes (LRC−, *n* = 16), HRA infants with negative ADOS outcomes (HRA−, *n* = 17), and HRA infants with positive ADOS outcomes (HRA+, *n* = 5).

### Coherence at 6 months of age

Analyses of data from 6-month-old infants revealed two significant main effects. Due to the nature of the statistical model used, effect sizes for significant model effects were calculated using Cohen’s *f*. There was a significant effect of hemisphere (F(1,44.78) = 5.42, *p*<0.03; Cohen’s *f* = 0.3), showing higher linear coherence in the right hemisphere as compared to the left. There was also a significant effect of condition (F(2,88) = 47.46, *p*<0.0001; Cohen’s *f* = 1.4), showing significantly higher linear coherence for both deviant conditions as compared to the standard (native vs. standard: t(45) = 8.90, *p*<0.0001, two-tailed, Cohen’s *d* = 1.3; non-native vs. standard: t(45) = 8.47, *p*<0.0001, two-tailed, Cohen’s *d* = 1.3), but no difference between deviant conditions (t(45) = 1.3, *p*<0.2; Cohen’s *d* = 0.2). There was no significant effect of group at this age. See table 3 for a complete summary of all results.

### Coherence at 12 months of age

Analyses of 12-month-old data produced two significant effects. Most notably, we found a significant main effect of group (F(1,42) = 7.77, *p*<0.005; Cohen’s *f* = 0.4), such that LRC infants displayed higher linear coherence that HRA infants (see Fig. 1). As in the 6-month data, there was a significant main effect of condition (F(2,84) = 77.33, *p*<0.0001; Cohen’s *f* = 1.8). For both groups, both deviant conditions elicited significantly more linear coherence than the standard condition (native vs. standard: t(43) = 12.79, *p*<0.0001, two-tailed, Cohen’s *d* = 1.8; non-native vs. standard: t(43) = 10.09, *p<*0.0001, two-tailed, Cohen’s *d* = 1.7), but no difference was found between the two deviant conditions (t(43) = 0.8, *p*<0.5, Cohen’s *d* = 0.1). The analyses did not reveal any significant difference between hemispheres, and no significant interactions among the factors. See table 3 for a complete summary of all results.

### Summary of coherence findings according to risk level

**Figure 2:**
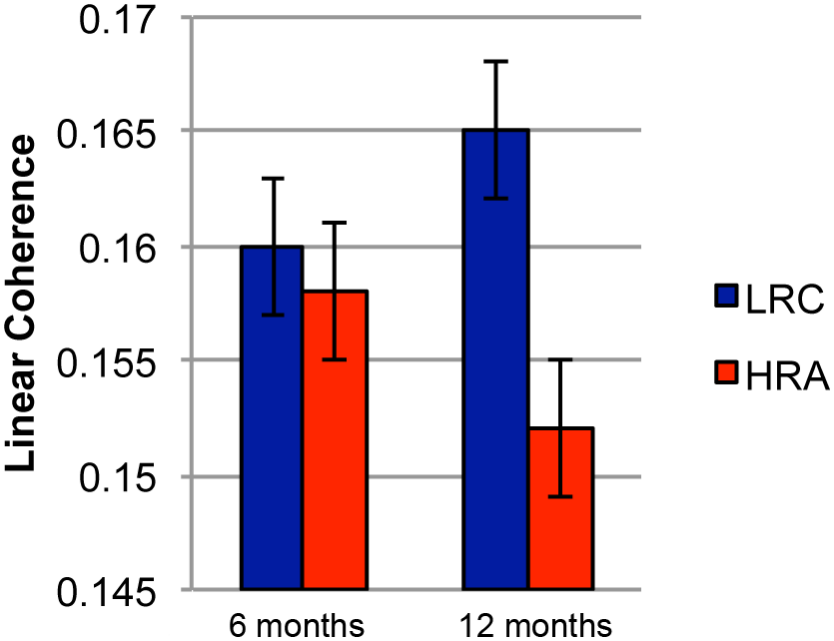
Average linear coherence for LRC (infants at low risk for ASD) and HRA (infants at risk for ASD) infants at 6 and 12 months. Error bars represent standard error.

To summarize, we found that differences between HRA and LRC were not present at 6 months of age, but by 12 months LRC infants showed overall greater coherence compared to HRC across all experimental conditions (see figure 2). Furthermore, at both ages and for both groups there was evidence of sensitivity to the deviant phonemes. Finally, 6-month olds showed higher linear coherence in the right hemisphere compared to the right, but no lateralization differences were found at 12 months of age.

### Differences according to ASD outcome–a preliminary analysis

**Figure 3:**
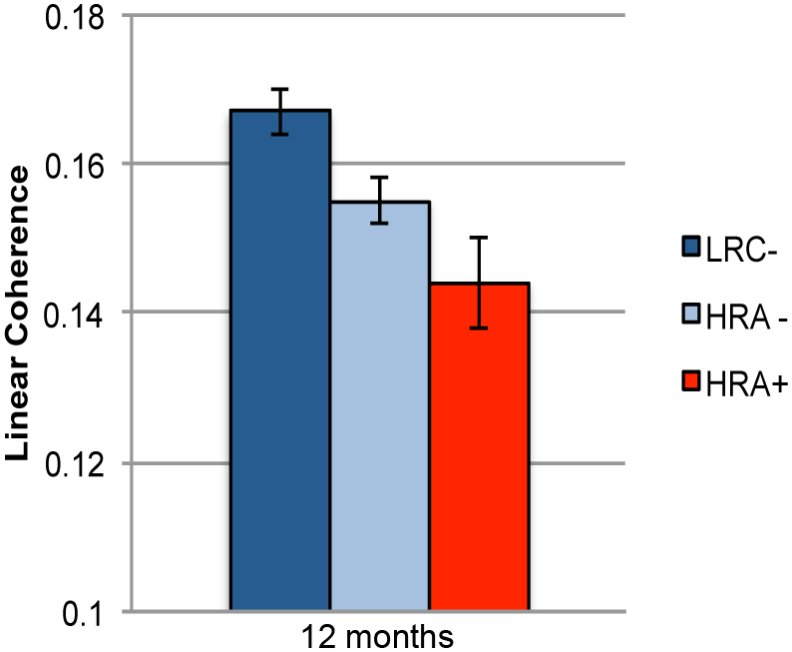
Average linear coherence at 12 months of age for LRC− (low risk infants who did not meet criteria for ASD at 36 months), HRA− (infants at risk for ASD who did not meet criteria for ASD at 36 months), HRA+ (infants at risk who met criteria for ASD at 36 months). Error bars represent standard error.

To determine whether the differences observed at 12 months between HRA and LRC infants are primarily associated with genetic risk for ASD or with the emergence of ASD we conducted a follow-up analysis on a subset of infants who had been assessed using the Autism Diagnostic Observation Schedule (ADOS) at 36 months of age. Out of the 44 infants (19 LRC and 25 HRA) who contributed data at 12 months of age, 38 of them had 36-month outcome data (16 LRC and 22 HRA). None of the 16 LRC infants met criteria for ASD on the ADOS, whereas, 5 out of the 22 HRA infants met ASD criteria on the ADOS. For this analysis, we used the same statistical approach described above with the exception that group, which is the between subjects factors, became a three-way variable: LRC infants with negative ADOS outcomes (LRC−; n = 16), HRA infants with negative ADOS outcomes (HRA−; n = 17), and HRA infants with positive ADOS outcomes (HRA+; n = 5). This analysis revealed a significant main effect of group (F(2,35) = 5.8; *p*<0.01; Cohen’s *f* = 0.5). Follow up t-tests revealed higher linear coherence in the LRC− group compared to both the HRA− and HRA+ groups (LRC− vs. HRA−: t(31) = 2.42, *p*<0.03, two-tailed, Cohen’s *d* = 0.9; LRC− vs. HRA+: t(19) = 2.67, *p*<0.02, two-tailed, Cohen’s *d* = 1.4). A marginally significant difference was observed between HRA− and HRA+ infants (t(20) = 1.74, *p*<0.1; two-tailed, *p*<0.05, one-tailed, Cohen’s *d* = 0.8) with higher coherence in the HRA− group As in the results presented in the previous section, there was a main effect of condition (F(2,70) = 52.2 *p*<0.0001; Cohen’s *f* = 1.5). Follow-up t-tests showed that both deviant conditions elicited significantly more linear coherence than the standard condition (native vs. standard: t(37) = 9.14, *p*<0.0001, two-tailed, Cohen’s *d* = 1.2; non-native vs. standard: t(37) = 8.5, *p<*0.0001, two-tailed, Cohen’s *d* = 1.1), but no difference was found between the two deviant conditions (t(37) = 0.6, *p*<0.6, two-tailed, Cohen’s *d* = 0.1). No other differences were observed between these three groups (see table 4 for full ANOVA results). See figure 3 for illustration of the observed effects.

## Discussion

The goal of the present study was to examine whether there are differences in functional connectivity between infants at high- and low- risk for ASD by the first year of life. First, our results show that by 12 months of age, infants at high-risk of developing ASD display significantly lower functional connectivity between frontal and parietal sites compared to infants at low-risk for ASD. Second, infants at high-risk for ASD showed lower functional connectivity compared to low-risk infants, irrespective of ASD outcome. Third, coherence appears to be lowest in those high-risk infants who go on to develop ASD, compared to high-risk infants who do not (see figure 3), but a larger sample is needed to confirm this result. Finally, our results did not show any group differences with regard to the sensitivity to native and non-native phonetic contrast or hemispheric lateralization.

The present results are consistent with a growing body of literature showing the emergence of risk-related differences in electrophysiological responses that emerge over the course of the first year of life [16], [37], [38]. More specifically, our findings of reduced functional connectivity associated with risk for ASD are consistent with recent studies of infants and toddlers that have used fNIRS [39], DTI [40], and fMRI [23]. Taken together, these results confirm the emerging trend toward atypical developmental patterns in measures of neural integration and anatomical connectivity associated with risk for ASD.

Our findings are the first to demonstrate the presence of reduced functional connectivity as indexed by linear coherence in gamma frequency activity in infants at high-risk for ASD. Furthermore, this is an endophenotype of ASD because reduced functional connectivity between our low-risk group and our high-risk group was present even after infants who went on to develop ASD were excluded from the analyses. Together with recent findings reporting abnormalities in white matter structures in the children with ASD and their unaffected siblings [41], our results provide evidence for a neural architecture that is both anatomically and functionally different from the early stages of development in individuals who are genetically vulnerable to ASD. It is likely that many qualitative and quantitative differences in neural connectivity can account for the heterogeneous phenotypic variations observed in individuals at risk for ASD. Our preliminary results suggest that quantitative differences in functional connectivity might be present between infants at-risk for ASD who later develop ASD and those who do not. This result is not surprising giving the strong evidence of reduced functional and anatomical connectivity in individuals ASD [42]. Nevertheless, it is remarkable that such differences can be detected as early as 12-months-of-age.

The stimuli used in this study derive from a paradigm designed to examine phonetic perception in relation to the perceptual content that infants are exposed to [27], [28]. A recent study carried out with the same sample of infants using ERP showed that both low- and high-risk infants displayed experience-dependent changes in their responses to native and non-native phonemes over the first year of life, such that by 12 months of age there were no differences in the ERP responses to a non-native phonetic contrast [28]. Here we demonstrate that linear coherence between frontal and parietal sites is a neural measure sensitive to differences between native and non-native contrasts in both 6-month and 12-month old infants irrespective of risk status, as evidenced by significant differences between the standard condition and both deviant conditions. This pattern is somewhat similar to some of the ERP findings by Seery and colleagues [28] in which, irrespective of risk status, at 6 months and 9 months the P150 amplitude in the frontal region showed a main effect of condition with the two deviant conditions different from the standard, but no differences between the deviants. Nevertheless, Seery and colleagues [28] found that at 12-months-of-age the P150 amplitude in the frontal region was no longer different between non-native deviant and standard conditions. It is important to point out that there are some key methodological differences between the two studies that could account for the differences in results at 12 months. First, linear coherence in the gamma frequency band and ERP used non-overlapping portions of the EEG signal: whereas we focused our analyses on frequencies from 30 to 50 Hz, the ERP signal contains information only from frequencies below 30 Hz. Second, linear coherence, as an index of synchronized activity across sites, is not dependent on morphological attributes of the localized EEG signal across trials, but rather on the relationship in phase and power of the EEG signal between 2 locations. In contrast, ERPs reflect localized activity and are primarily sensitive to the presence of specific morphological characteristics within the EEG signal that are consistent across experimental trials. Third, in Seery et al. [28] these effects were found only in the frontal region, whereas the linear coherence reported on here assesses the connection between frontal and parietal regions.

Previous electrophysiological research on infant siblings has also found evidence of differences in hemispheric lateralization in ERP responses to speech sounds between low- and high-risk infants [28]. Similarly atypical patterns of hemispheric lateralization have also been found using fMRI in tasks that involved language processing [43], [44]. In the present study we failed to find any differences in hemispheric lateralization of linear coherence, regardless of risk status or ASD outcome. As discussed above, linear coherence is an index of synchronized activity between regions and does not reflect the absolute amount of activation in response to specific stimuli, in contrast to ERP and fMRI. As such, it is possible that over the first year of life the left and right hemisphere networks sampled by the electrodes chosen in this study are comparably synchronous, irrespective of any differences in absolute activation.

### Limitations

The present study has several limitations, primarily related to the nature of the infant sibling studies methodology. First, the combination of the available sample size and the fact that not all infants contributed data points at all ages deemed these data ill-suited for longitudinal analyses that could have shed light to developmental trajectories, Second, given that only a small number of infants met ASD criteria at 36 months (5 infants), our analyses cannot speak directly to the potential for reduced functional connectivity as a neurobiological marker of ASD. Third, it has been suggested that high frequency signals in the EEG are vulnerable to myogenic artifacts [45] and eye movement artifacts [46]. Nevertheless, it is unlikely that these artifacts would have any group- or condition-specific effects.

### Conclusions

To conclude, the present study demonstrated that reduced functional connectivity during speech processing is a trait associated with family risk status, and can therefore be considered an endophenotype. While the present study cannot speak directly to the relationship between clinical outcome and functional connectivity, it provides preliminary evidence suggesting that functional connectivity at 12 months is lowest in those infants who do go onto develop ASD. This provides further evidence that ASD is broadly characterized by differences in neural integration. In the future it will be important to determine how the emergence of atypical task-related functional connectivity by the first year of life contributes to a cumulative risk model, which can lead to further understanding of the factors that lead to the development of an ASD diagnosis [12], [47].

## References

[1] American Psychiatric Association. (2013) Diagnostic and statistical manual of mental disorders (5th ed.). Arlington, VA: American Psychiatric Publishing.

[2] Tager-Flusberg H, Joseph RM (2003) Identifying neurocognitive phenotypes in autism. Philos Trans R Soc Lond B Biol Sci 358: 303–14. 12639328 10.1098/rstb.2002.1198 PMC1201482

[3] Amaral DG, Schumann CM, Nordahl CW (2008) Neuroanatomy of autism. Trends Neurosci 31: 137–45. 18258309 10.1016/j.tins.2007.12.005

[4] Murdock JD, State MW (2013) Recent development in the genetics of autism spectrum disorders. Curr Opin in Genet Dev 23: 310–15. 23537858 10.1016/j.gde.2013.02.003

[5] Geschwind DH, Levitt P (2007) Autism spectrum disorders: Developmental disconnection syndromes. Curr Opin Neurobiol 17: 103–11. 17275283 10.1016/j.conb.2007.01.009

[6] Uhlhaas PJ, Singer W (2006) Neural synchrony in brain disorders: Relevance for cognitive dysfunctions and pathophysiology. Neuron 52: 155–68. 17015233 10.1016/j.neuron.2006.09.020

[7] Brock J, Brown CC, Boucher J, Rippon G (2002) The temporal binding deficit hypothesis of autism. Dev Psychopathol 14: 209–24. 12030688 10.1017/s0954579402002018

[8] Zwaigenbaum L, Thurm A, Stone W, Baranek G, Bryson S, et al (2007) Studying the emergence of autism spectrum disorders in high-risk infants: Methodological and practical issues. J Autism Dev Disord 37: 466–80. 16897376 10.1007/s10803-006-0179-x

[9] Zwaigenbaum L, Bryson S, Lord C, Rogers S, Carter A, et al (2009) Clinical assessment and management of toddlers with suspected autism spectrum disorder: Insights from studies of high-risk infants. Pediatrics 123: 1383–91. 19403506 10.1542/peds.2008-1606 PMC2833286

[10] Rogers SJ (2009) What are infant siblings teaching us about autism in infancy? Autism Res 2: 125–37. 19582867 10.1002/aur.81 PMC2791538

[11] Ozonoff S, Young GS, Carter A, Messinger D, Yirmiya N, et al (2011) Recurrence risk for autism spectrum disorders: A baby siblings research consortium study. Pediatrics 128: 488–95. 10.1542/peds.2010-2825 PMC3164092 21844053

[12] Jones E, Gliga T, Bedford R, Charman T, Johnson MH (2014) Developmental pathways to autism: A review of prospective studies of infants at risk. Neuroscience and Biobehavioral Reviews 39: 1–33. 24361967 10.1016/j.neubiorev.2013.12.001 PMC3969297

[13] Gottesman II, Gould TD (2003) The endophenotype concept in psychiatry: Etymology and strategic intentions. Am J Psychiatry 160: 636–45. 12668349 10.1176/appi.ajp.160.4.636

[14] Viding E, Blakemore SJ (2007) Endophenotype approach to developmental psychology: Implications for Autism research. Behav Genet 37: 51–60. 16988798 10.1007/s10519-006-9105-4

[15] Elsabbagh M, Volein A, Csibra G, Holmboe K, Garwood H, et al (2009) Neural correlates of eye gaze processing in the infant broader autism phenotype. Biol Psychiatry 65: 31–8. 19064038 10.1016/j.biopsych.2008.09.034

[16] Tierney AL, Gabard-Durnam L, Vogel-Farley V, Tager-Flusberg H, Nelson CA (2012) Developmental trajectories of resting EEG power: An endophenotype of autism spectrum disorder. PLoS ONE 7: e39127. 22745707 10.1371/journal.pone.0039127 PMC3380047

[17] Catarino A, Andrade A, Churches O, Wagner AP, Baron-Cohen S, et al (2013) Task-related functional connectivity in autism spectrum conditions: An EEG study using wavelet transform coherence. Mol Autism 4: 1–14. 23311570 10.1186/2040-2392-4-1 PMC3558480

[18] Coben R, Clarke AR, Hudspeth W, Barry RJ (2008) EEG power and coherence in autistic spectrum disorder. Clin Neurophysiol 119: 1002–9. 18331812 10.1016/j.clinph.2008.01.013

[19] Murias M, Webb SJ, Greenson J, Dawson G (2007) Resting state cortical connectivity reflected in EEG coherence in individuals with autism. Biol Psychiatry 62: 270–3. 17336944 10.1016/j.biopsych.2006.11.012 PMC2001237

[20] Peters JM, Taquet M, Vega C, Jeste SS, Fernández IS, et al (2013) Brain functional networks in syndromic and non-syndromic autism: A graph theoretical study of EEG connectivity. BMC Med 11: 54–70. 23445896 10.1186/1741-7015-11-54 PMC3626634

[21] Lindgren KA, Folstein SE, Tomblin JB, Tager-Flusberg H (2009) Language and reading abilities of children with autism spectrum disorders and specific language impairment and their first-degree relatives. Autism Res 2 2: 22–38. 10.1002/aur.63 PMC2806306 19358305

[22] Toth K, Dawson G, Meltzoff AN, Greenson J, Fein D (2007) Early social, imitation, play, and language abilities of young non-autistic siblings of children with autism. J Autism Dev Disord 37: 145–57. 17216560 10.1007/s10803-006-0336-2 PMC2259442

[23] Dinstein I, Pierce K, Eyler L, Solso S, Malach R, et al (2011) Disrupted neural synchronization in toddlers with autism. Neuron 70: 1218–25. 21689606 10.1016/j.neuron.2011.04.018 PMC3119852

[24] Harris GJ, Chabris CF, Clark J, Urban T, Aharon I, et al (2006) Brain activation during semantic processing in autism spectrum disorders via functional magnetic resonance imaging. Brain Cogn 61: 54–68. 16473449 10.1016/j.bandc.2005.12.015

[25] Just MA, Cherkassky VL, Keller TA, Minshew NJ (2004) Cortical activation and synchronization during sentence comprehension in high-functioning autism: Evidence of underconnectivity. Brain 127: 1811–21. 15215213 10.1093/brain/awh199

[26] Knaus TA, Silver AM, Lindgren KA, Hadjikhani N, Tager-Flusberg H (2008) FMRI activation during a language task in adolescents with ASD. J Int Neuropsychol Soc 14: 967–79. 18954477 10.1017/S1355617708081216 PMC2747321

[27] Rivera-Gaxiola M, Silva-Pereyra J, Kuhl PK (2005) Brain potentials to native and non-native speech contrasts in 7- and 11-month-old American infants. Dev Sci 8: 162–72. 15720374 10.1111/j.1467-7687.2005.00403.x

[28] Seery AM, Vogel-Farley V, Tager-Flusberg H, Nelson CA (2012) Atypical lateralization of ERP response to native and non-native speech in infants at risk for autism spectrum disorder. Dev Cogn Neurosci 5C: 10–24. 10.1016/j.dcn.2012.11.007 PMC3676696 23287023

[29] Rutter M, Bailey A, Lord C (2003) Social Communication Questionnaire. In: Los Angeles, CA: Western Psychological Services.

[30] Lord C, Risi S, Lambrecht L, Cook EHJ, Leventhal BL, et al (2000) The autism diagnostic observation schedule-generic: A standard measure of social and communication deficits associated with the spectrum of autism. J Autism Dev Disord 30: 205–23. 11055457

[31] Mullen E (1995) *Mullen Scales of Early Learning*. Circle Pines, MN: American Guidance Services Inc.

[32] Kawahara H, Masuda-Kasuse I, de Cheveigne A (1999) Restructuring speech representations using a pitch-adaptive time-frequency smoothing and an instantaneous-frequency-based F0 extraction: Possible role of a repetitive structure in sounds. Speech Commun 27: 187–207.

[33] Kuhl P, Williams KA, Lacerda F, Stevens KN, Lindblom B (1992) Linguistic experience alters phonetic perception in infants by 6 months of age. Science 255: 606–608. 1736364 10.1126/science.1736364

[34] Cheour M, Ceponiene R, Lehtokoski A, Luuk A, Allik J, et al (1998) Development of language-specific phoneme representations in the infant brain. Nat Neurosci 1: 351–353. 10196522 10.1038/1561

[35] Hoehl S, Wahl S (2012) Recording infant ERP data for cognitive research. Dev Neuropsychol 37: 187–209. 22545658 10.1080/87565641.2011.627958

[36] Delorme A, Makeig S (2004) EEGLAB: An open source toolbox for analysis of single-trial EEG dynamics including independent component analysis. J Neurosci Methods 134: 9–21. 15102499 10.1016/j.jneumeth.2003.10.009

[37] Bosl W, Tierney A, Tager-Flusberg H, Nelson C (2011) EEG complexity as a biomarker for autism spectrum disorder risk. BMC Med 9: 18–32. 21342500 10.1186/1741-7015-9-18 PMC3050760

[38] Gabard-Durnam L, Tierney AL, Vogel-Farley V, Tager-Flusberg H, Nelson CA (2013) Alpha asymmetry in infants at risk for autism spectrum disorders. J Autism Dev Disord: 1–8. 10.1007/s10803-013-1926-4 PMC3938993 23989937

[39] Keehn B, Wagner JB, Tager-Flusberg H, Nelson CA (2013) Functional connectivity in the first year of life in infants at-risk for autism: A preliminary near-infrared spectroscopy study. Front Hum Neurosci 7: 444. 23964223 10.3389/fnhum.2013.00444 PMC3734360

[40] Wolff JJ, Gu H, Gerig G, Elison JT, Styner M, et al (2011) Differences in white matter fiber tract development present from 6 to 24 months in infants with autism. Am J Psychiatry 169: 589–600. 10.1176/appi.ajp.2011.11091447 PMC3377782 22362397

[41] Barnea-Goraly N, Lotspeich LJ, Reiss AL (2010) Similar white matter aberrations in children with autism and their unaffected siblings: A diffusion tensor imaging study using tract-based spatial statistics. Arch Gen Psychiatry 67: 1052–60. 20921121 10.1001/archgenpsychiatry.2010.123

[42] Just MA, Keller TA, Malave VL, Kana RK, Varma S (2012) Autism as a neural systems disorder: A theory of frontal-posterior underconnectivity. Neurosci Biobehav 36: 1292–313. 10.1016/j.neubiorev.2012.02.007 PMC3341852 22353426

[43] Eyler LT, Pierce K, Courchesne E (2012) A failure of left temporal cortex to specialize for language is an early emerging and fundamental property of autism. Brain 135: 949–60. 22350062 10.1093/brain/awr364 PMC3286331

[44] Redcay E, Courchesne E (2008) Deviant functional magnetic resonance imaging patterns of brain activity to speech in 2-3-year-old children with autism spectrum disorder. Biol Psychiatry 64: 589–98. 18672231 10.1016/j.biopsych.2008.05.020 PMC2879340

[45] Goncharova II, McFarland DJ, Vaughan TM, Wolpaw JR (2003) EMG contamination of EEG: Spectral and topographical characteristics. Clin Neurophysiol 114: 1580–93. 12948787 10.1016/s1388-2457(03)00093-2

[46] Yuval-Greenberg S, Tomer O, Keren AS, Nelken I, Deouell LY (2008) Transient induced gamma-band response in EEG as a manifestation of miniature saccades. Neuron 58: 429–41. 18466752 10.1016/j.neuron.2008.03.027

[47] Tager-Flusberg H (2010) The origins of social impairments in autism spectrum disorder: Studies of infants at risk. Neural Netw 2010 23: 1072–6. 10.1016/j.neunet.2010.07.008 PMC2956843 20800990

